# Identification and Characterization of an Isoform Antifreeze Protein from the Antarctic Marine Diatom, *Chaetoceros neogracile* and Suggestion of the Core Region

**DOI:** 10.3390/md15100318

**Published:** 2017-10-18

**Authors:** Minjae Kim, Yunho Gwak, Woongsic Jung, EonSeon Jin

**Affiliations:** 1 Department of Life Science, College of Natural Sciences, Hanyang University, Seoul 133-791, Korea; sciencekor89@gmail.com (M.K.); firsthero@macrogen.com (Y.G.); 2 Division of Polar Life Science, Korea Polar Research Institute, KIOST, Incheon 406-840, Korea

**Keywords:** Antarctic marine diatom, *Chaetoceros neogracile*, isoform antifreeze protein, antifreeze activity, 3D-structure modeling

## Abstract

Antifreeze proteins (AFPs) protecting the cells against freezing are produced in response to extremely low temperatures in diverse psychrophilic organisms, and they are encoded by multiple gene families. The AFP of Antarctic marine diatom *Chaetoceros neogracile* is reported in our previous research, but like other microalgae, was considered to probably have additional genes coding AFPs. In this paper, we reported the cloning and characterization of additional AFP gene from *C. neogracile* (*Cn-isoAFP*). *Cn-isoAFP* protein is 74.6% identical to the previously reported Cn-AFP. The promoter sequence of *Cn-isoAFP* contains environmental stress responsive elements for cold, thermal, and high light conditions. *Cn-isoAFP* transcription levels increased dramatically when cells were exposed to freezing (−20 °C), thermal (10 °C), or high light (600 μmol photon m^−2^ s^−1^) stresses. The thermal hysteresis (TH) activity of recombinant Cn-isoAFP was 0.8 °C at a protein concentration of 5 mg/mL. Results from homology modeling and TH activity analysis of site-directed mutant proteins elucidated AFP mechanism to be a result of flatness of B-face maintained via hydrophobic interactions.

## 1. Introduction

Antifreeze proteins (AFPs) are found in various organisms such as fishes, insects, plants, microalgae and even bacteria living in Arctic and Antarctic regions [1,2,3]. AFPs are able to decrease the freezing point of the fluid below its melting point and inhibit ice-recrystallization, which helps in survival of the organism at extremely low temperatures [4]. The basic mechanisms of the protein functions have been studied through structure predictions using protein modeling programs or structure identification using X-ray crystallography [5,6,7,8,9,10,11]. Like multicellular organisms, microalgal AFPs such as *Navicula glaciei* [12], *Fragilariopsis cylindrus* [13], *Chlamydomonas* sp. [14], *Chloromonas* sp. [15], and *Fragilariopsis* sp. [16], have also been studied using diverse analytic approaches. Interestingly, many microalgal AFPs are encoded with multiple genes, similar to other organisms [17,18]. The presence of multiple gene families of AFPs supports the notion that AFPs are required for psychrophilic organisms to adapt and survive in extremely low-temperature environments.

Based on previous research, the mechanism of AFPs has been considered as that an ice crystal binding activity and a reducing the freezing temperature are most probably due to an absorption-inhibition mechanism [4,19,20,21,22]. The “anchored clathrate water” mechanism was proposed to explain *Marinomonas primoryensis* AFP(MpAFP)-binding to the ice lattice [23]. MpAFP arranges water molecules into an ice-like lattice on the ice-binding surface (IBS). The gamma-methyl groups of Thr residues on the IBS are enclosed by water molecules. This type of “cage” is anchored on the IBS by hydrogen bonding to the nitrogen in the main chain and hydroxyl functional groups in the side chain of Thr. The anchored clathrate water mechanism can be explained as the organized water molecules anchored to the protein by hydrogen bonds such as ice-like lattice [23]. Although there are many reports about characterization of AFPs at a gene to protein level and even its industrial applications [24], the mechanism of AFPs on a biochemistry and biophysics level has still not been fully discovered.

The AFP of *Chaetoceros neogracile* (Cn-AFP), an Antarctic marine diatom, has been reported [25]. The expression of *Cn-AFP* was sharply induced by various environmental stresses, and its 5′ upstream sequence was predicted to contain light, cold, and heat shock-responsive elements [26]. Similar to *Cn-AFP*, other psychrophilic diatom AFPs also show dynamic expression in response to environmental stresses like high salt- and low-temperature culture conditions [16]. The structure of Cn-AFP was predicted using 3D modeling program and was comparable to the protein structure of AFPs from a fungus and Arctic yeast. However, we considered that *C. neogracile* also has additional genes to maintain more organically their intracellular environment from extremely low temperature like other diatom AFPs [26].

In this paper, we cloned and sequenced an additional AFP gene from *C. neogracile* (*Cn-isoAFP*) and compared it to the AFPs of other psychrophilic organisms. Promoter regions and functional motifs were analyzed by prediction programs. To confirm the promoter results, we examined the expression of *Cn-isoAFP* under various stress conditions. In addition, we propose a protein structure for *Cn-isoAFP* and the mechanisms underlying its antifreeze activity based on homology modeling and site-directed mutagenesis.

## 2. Results

### 2.1. Cloning and Identification of the C. neogracile Isoform AFP

The sequence of a *C. neogracile* AFP gene (*Cn-AFP*) and its expression in response to cold stress has been reported previously [25]. Based on this previously identified *Cn-AFP* sequence, another AFP of *C. neogracile* was amplified with degenerate primers, and the full sequence was obtained by 5′ DNA walking and 3′-RACE PCR. The degenerate primers were designed to target the domains with high sequence conservation among psychrophilic microalgal AFPs at the amino acid level. The *Cn-isoAFP* ORF was 855 bp long, encoding a 284 amino acid protein. The predicted molecular weight of the protein was 29.4 kDa. The signal peptide of *Cn-isoAFP* protein was identified using SignalP [27]; it was 31 amino acids in length and contained a glycosylation and a myristoylation site (Figure S1). The molecular mass of mature *Cn-isoAFP* without a signal sequence was about 25.9 kDa. The gene sequence of *Cn-isoAFP* was 74.8% identical to that of *Cn-AFP* (ACU09498) (Figure S2). Southern blot analysis using the *Cn-isoAFP* gene as a probe showed the possibility that *C. neogracile* might have more than two *AFP* genes, besides the *Cn-isoAFP* and the previously reported *Cn-AFP* (Figure S3).

### 2.2. Phylogenetic Analysis of Cn-isoAFP Using Multiple Alignment

*Cn-isoAFP* showed high sequence similarity to the AFPs of other psychrophilic diatoms, fungi, and bacteria (Figure S4). The *Cn-isoAFP* exhibited high identity (57.4%) to the Antarctic ice diatom *N. glaciei* AFP. *Cn-isoAFP* also showed high sequence identity to the AFP genes of *F. curta* (sea ice diatom, 38.8%), *T. ishikariensis* (snow mold, 42.4%), *Colwellia* sp. (Antarctic sea ice bacteria, 39.1%), and *P. ingrahamii* (psychrophilic bacteria, 41.5%). However, *Cn-isoAFP* exhibited very low similarity to the genes of the psychrophilic green microalgae *Chlamydomonas* sp. (10.5%). Sequence alignment and phylogenetic analysis showed that the *Cn-AFP* and *Cn-isoAFP* genes are evolutionarily close to the *N. glaciei* AFP gene (Figures S4 and S5). *Cn-isoAFP* and Cn-AFP are related to other eukaryotic AFPs (snow mold, other ice diatom) and bacterial AFPs. However, *Cn-isoAFP* is evolutionarily distinct from plant AFPs, ice recrystallization inhibition proteins (IRIPs), and green microalgal AFPs (Figure S5).

### 2.3. In Silico Analysis of the Cn-isoAFP Promoter

**Figure 1:**
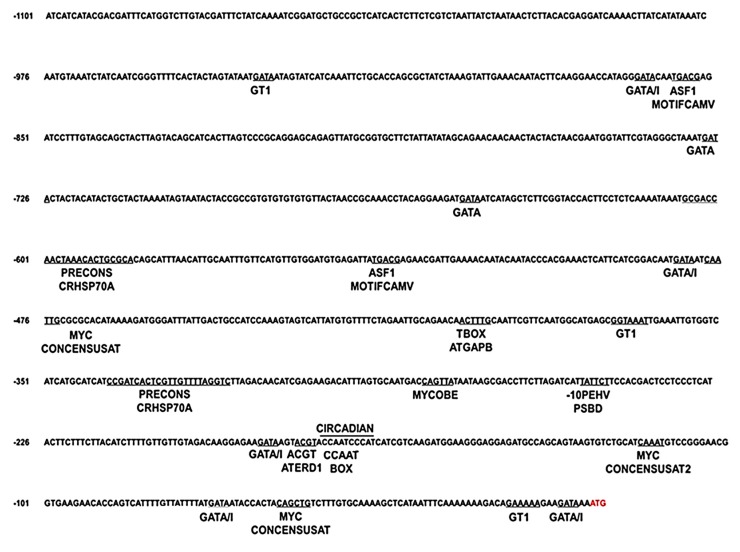
In silico sequence analysis of the *Cn-isoAFP* promoter and the putative transcription factor binding sites. The 5′ upstream of the *Cn-isoAFP* gene contained various environmental stress responsive elements such as, high light, dehydration, heat and cold shock. All motifs were underlined (or upperlined) and the name of responsive elements are indicated under (or above) the line. The predicted TATA box and CAAT box were solid underlined. The red color letters show the transcription start site.

The 5′-upstream sequence of *Cn-isoAFP* was identified and analyzed using promoter database programs to investigate the potential physiological roles of this protein [28,29,30]. Various putative transcription factor binding sites responsive to environmental stresses and TATA and CCAAT boxes were identified (Figure 1). MYCCONSENSUSAT [31], which is a cold-responsive element in higher plants, was present in the *Cn-isoAFP* promoter sequence. Furthermore, light-responsive elements such as GATA boxes [32], I BOX [33], 10PEHVPSBD [34], ASF1MOTIFCAMV [35], GT1CONSENSUS [35], and TBOXATGAPB [36] were also present. Since freezing induces dehydration and water stress, dehydration and water stress-responsive motifs, ACGTATERD1 [37], MYBCORE [38], and MYCCONSENSUSAT [31] , were found in the *Cn-AFP* promoter. The HSP70A promoter of *Chlamydomonas* (PRECONSCRHSP70A) [39] and transcriptional enhancement of circadian control CIRCADIALELHC [40] motifs were also found in the *Cn-isoAFP* promoter sequence.

### 2.4. Gene Expression Analysis of Cn-isoAFP in Response to Stress Conditions

To confirm the promoter analysis results, we analyzed the expression of *Cn-isoAFP* under freezing (−20 °C), thermal (10 °C), and high light (HL, 600 μmol photon m^−2^ s^−1^) stress conditions.

**Figure 2:**
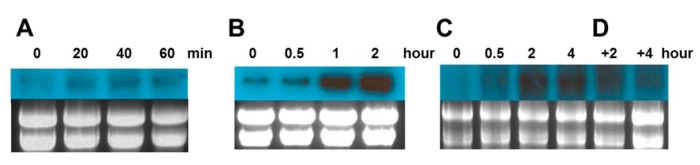
Gene expression analysis *Cn-isoAFP* under various conditions on environmental stress. (**A**), Freezing (−20 °C) stress; (**B**), Thermal (10 °C) stress; (**C**), High light (600 μmol photon m^−2^ s^−1^) stress; (**D**), 4 h high light stressed cells were transferred to the normal condition. The upper panels are *Cn-isoAFP* genes and lower panels are EtBr stained RNA as a loading control.

To impose freezing stress, cells were kept at −20 °C for 20, 40, and 60 min, respectively. The cell culture medium began to freeze at 20 min and was completely frozen after 60 min. *Cn-isoAFP* transcription levels were up-regulated in response to freezing stress 20 min after treatment (Figure 2A). Under thermal stress (10 °C), the *Cn-isoAFP* gene was rapidly induced in 1 h compared to baseline (Figure 2B). Under HL stress (600 μmol photon m^−2^ s^−1^), *Cn-isoAFP* transcription levels increased rapidly within 1 h, and the expression was continuously induced for up to 4 h (Figure 2C). When the 4 h HL-stressed cells were transferred to normal culture conditions, *Cn-isoAFP* transcription levels decreased dramatically (Figure 2D).

### 2.5. Homology Modeling of the Structure of Cn-isoAFP

**Figure 3:**
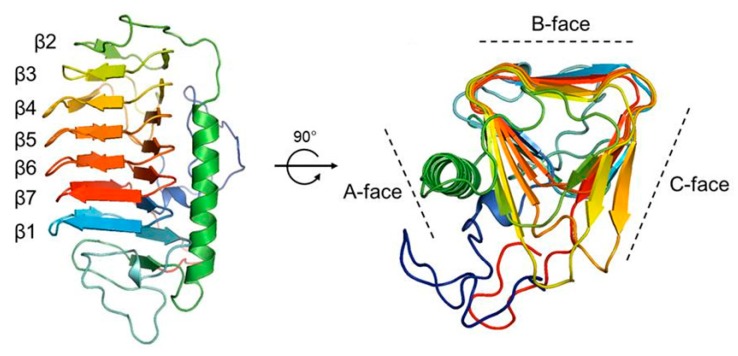
The 3D-structure of *Cn-isoAFP*. The AFP of *T. ishikeriensis* was used as the template of homology modeling. *Cn-isoAFP* was composed of three faces showing a triangle cross section. The A-face of *Cn-isoAFP* was covered by an α-helix and the B-face formed a relatively flat surface predicted as IBS.

*Cn-isoAFP* is composed of one *α*-helix and seven *β*-loops (Figure 3). The tertiary structure of Cn-isoAFP was predicted by Phyre2, Modeller v9.12 and PyMOL. *T. ishikariensis* AFP6 (TisAFP6; PDB ID, 3VN3) was used as an amino acid template for protein modeling of *Cn-isoAFP*. *Cn-isoAFP* has 46% structural identity with TisAFP6. The results showed that the structure of *Cn-isoAFP* had high similarity to that of TisAFP6 with 100% confidence. In addition, the protein structures of each mutated *Cn-isoAFP* were modeled and predicted using the same homology modeling methods described for *Cn-isoAFP*.

**Figure 4:**
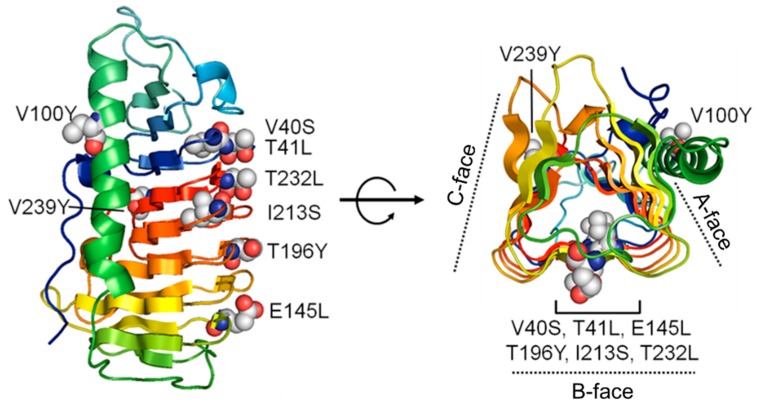
Site-directed mutagenesis to reveal the ice-binding sites of Cn-isoAFP. The single amino acid substitutions to Tyr were performed on A-face and C-face. In addition, six sites for replacement with Tyr, Ser and Leu were demonstrated on the image showing the Cn-isoAFP structure.

*Cn-isoAFP* had three faces located on side surfaces of β-helix with a triangle cross-section (Figure 3). The A-face of *Cn-isoAFP* was covered by α-helix, and the B-face forms a relatively flat face. The B-face of *Cn-isoAFP* formed one right-handed β-helix with seven loops. The β1 loop near the N-terminus was on the β7 loop, which was located on the C-terminal region. Seven helical loops were formed in the following irregular order: β1-β7-β6-β5-β4-β3-β2 (Figure 3). Six mutant proteins were produced in order to identify IBS (V100Y, T196Y and V239Y) and amino acids interacting with ice lattice oxygen atoms (T41L, E145L and T232L). In addition, two mutants were generated to demonstrate the importance of the hydrophobic core (V40S and I213S). The mutated sites are indicated by balls in Figure 4.

### 2.6. The Antifreeze Activity of Cn-isoAFP and Its Mutant Proteins

**Figure 5:**
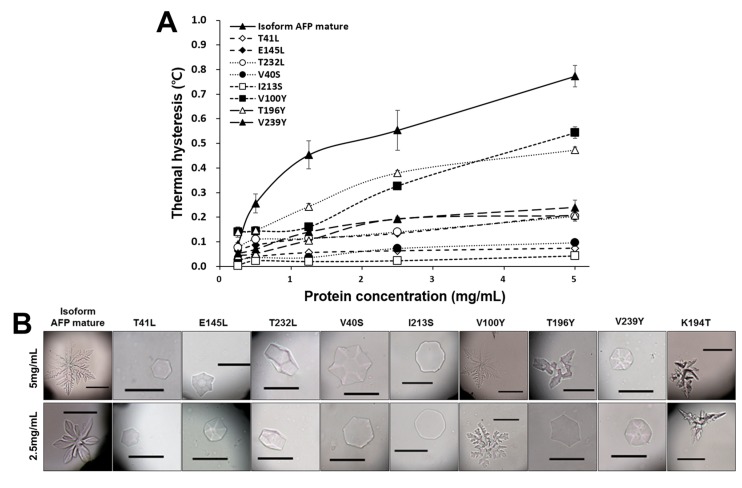
Antifreeze activities of Cn-isoAFP under various concentrations of the proteins. (**A**), thermal hysteresis activity of Cn-isoAFP and its site-directed mutants; (**B**), ice crystal growth morphology of Cn-isoAFP and its site-directed mutants. The scale bar indicated 100 μm.

Recombinant *Cn-isoAFP* protein was assayed for thermal hysteresis (TH) activity and its effect on ice crystal growth and morphology. Pre-mature *Cn-isoAFP* with the signal peptide did not show any TH activity and only induced minor morphological changes in ice crystals (data not shown). The shapes of the ice crystals formed in the presence of mature Cn-isoAFP (without a signal peptide sequence) were investigated (Figure 5B). The mature form of recombinant *Cn-isoAFP* yielded hexagonal ice crystals at a protein concentration of 0.25 mg/mL. All ice crystals formed in the presence of *Cn-isoAFP* exhibited a “burst growth” at high protein concentrations (above 0.5 mg/mL). The shapes of ice crystals became sharper at higher protein concentrations. The maximum TH activity of Cn-*isoAFP* was 0.8 °C at a concentration of 5 mg/mL.

The TH activities of all mutagenic *Cn-isoAFPs* were lower than the wild-type *Cn-isoAFP*, and the shapes that ice crystals adopted in the presence of these proteins fell into three categories: (1) hexagonal, (2) bipyramidal-like, or (3) circular (Figure 5 and Figure S6). The V100Y mutant protein yielded ice crystal forms similar to wild-type *Cn-isoAFP*. Ice crystals grown in the presence of Thr-substituted mutant proteins (T41L, T196Y, and T232L) had a hexagonal shape. The ice crystal morphologies induced by growth in the presence of V40S, E145L, and V239Y mutant proteins were bipyramidal-like. In particular, I213S yielded ice crystals with a circular shape, which is characteristic of complete loss of antifreeze activity. The TH activity of V100Y was half that of *Cn-isoAFP*, while the TH activities of E145L, T232L, T196Y, and V239Y were one-fourth that of *Cn-isoAFP* (Figure 5A). V40S, T41L, and I213S had a TH activity less than 0.1 °C, which indicated complete loss of TH activity.

### 2.7. Modeling of Cn-isoAFP Mutants Generated by Site-Directed Mutagenesis

**Figure 6:**
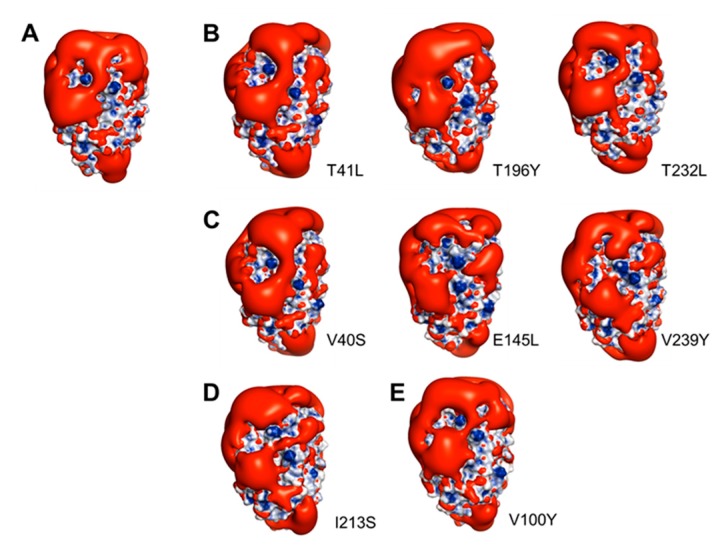
Distribution of negative isosurfaces overlapped on electropotential surfaces analysis from applied Poisson-Boltzmann solver tools. Colors of blue, white and red indicated positive, neutral and negative charges, respectively. Negative isosurfaces were represented to the red smooth surfaces. All of the Cn-isoAFP surfaces were illustrated to the B-faces. (**A**), Cn-isoAFP; (**B**), mutagenic proteins showing hexagonal ice crystals (T41L, T196Y, and T232L); (**C**), mutagenic proteins showing bipyramidal-like ice crystals (V40S, E145L, and V239Y); (**D**), mutagenic protein showing circular disc ice crystals (I213S, complete loss of antifreeze activity); (**E**), mutagenic protein showing similar morphology of ice crystals to Cn-isoAFP (V100Y).

The B-face of TisAFP6, which we used as a template for modeling *Cn-isoAFP*, was proposed to be an ice-interacting surface based on the results obtained for mutant TisAFP6 proteins generated by site-directed mutagenesis (Figure 4). We generated eight mutated *Cn-isoAFPs*. After homology modeling using Modeller and PyMOL, individual amino acids were substituted with leucine, tyrosine, or serine to predict crucial interactions for formation of an IBS. All predicted protein structures were assessed to be reliable models based on Ramachandran plot analysis (97.3% Ramachandran-favored, 2.3% Ramachandran-allowed, and 0.5% rotamer outliers) (data not shown) [41]. No significant topological modification of parts of functional groups was detected (data not shown). In addition to topological analyses of specific amino acids (Figure 6), the electrostatic characteristics of the protein surfaces of *Cn-isoAFP* and mutant proteins were investigated using the adaptive Poisson-Boltzmann solver [42]. The B-face of the V100Y mutant protein showed electrostatic potentials similar to that of *Cn-isoAFP*. The overall distribution of electrostatic potential fields on the B-face of V40S, T41L, E145L, T196Y, T232L, and V239Y was characterized by a similar proportion of positive and negative charges to that of *Cn-isoAFP*. In contrast, I213S mutated *Cn-isoAFP* had a large neutral electrostatic potential at the center of the B-face. In addition, a negative electrostatic potential field was found to be present across the B-face of I213S.

## 3. Discussion

Cn-AFP, an antifreeze protein from an Antarctic marine diatom, has been studied from a physiological and structural perspective [25,26]. Like other AFPs which have a multiple gene family [12,13,14,15,16,17,18], it was expected that *C. neogracile* would possess additional isoform AFPs. In this study, we reported a new isoform of *C. neogracile* AFP gene (*Cn-isoAFP*) and characterized the protein based on biochemical and physiological analysis. The *Cn-isoAFP* was strongly expressed under thermal and high light stresses, and the pattern was almost similar to Cn-AFP. In addition, our comparative results of protein functions and structural analysis between *Cn-isoAFP* and its site-directed mutants, showed that B-face of *Cn-isoAFP* is an ice-binding surface (IBS).

The presence of additional AFP genes in *C. neogracile* was predicted based on Southern blot analysis (Figure S3). We cloned the putative sequence using degenerate PCR method and compared it to the previously reported Cn-AFP. The gene sequence of *Cn-isoAFP* was 74.8% identical to that of *Cn-AFP* [25]. Alignment and phylogenetic analysis of the amino acid sequences of AFPs revealed that Cn-isoAFP is closely related to AFPs from sea ice diatoms and psychrophilic microorganisms (Figures S4 and S5). Surprisingly, the winter flounder genome encodes 30–50 AFPs [43,44], and the wolffish has more than 80 AFPs [45]. Thus, the presence of multiple AFP genes suggests that they play an important role in survival in a freezing environment. These results also indicate a possibility of multiple gene encoded AFP for *C. neogracile*.

Gene expression analysis data demonstrated that *Cn-isoAFP* was upregulated not only in low temperature stress condition, but also other stress inducing factors (Figure 2). Many light and temperature-responsive motifs were detected by transcription factor analysis in the promoter region of *Cn-isoAFP* (Figure 1). The up-regulated pattern of expression of *Cn-isoAFP* in response to environmental stress condition was similar to that of *Cn-AFP* [26]. Therefore, *Cn-isoAFP* seemed to play an important role in both antifreeze activity and resistance to environmental fluctuations.

Generally, AFPs inhibit ice crystal growth and lower the freezing point via an adsorption-inhibition mechanism, called thermal hysteresis (TH) [4,19]. Measurement of TH is the best method for quantitative assessment of antifreeze activity [46]. We obtained a maximum TH value of 0.8 °C for 5 mg/mL *Cn-isoAFP* (Figure 5A). This TH value is lower than the TH value of Cn-AFP (about 1.2 °C for 5 mg/mL, [26]) but much higher than that of TisAFP6 (0.2 °C for 8 mg/mL, [5]). Generally the fish TH value is about 1.0 °C at a protein concentration of 10 mg/mL [47] and that of AFP8 of a snow mold (*T. ishikariensis*) is 1.9 °C [48]. Hence antifreeze activity of *Cn-isoAFP* is not significantly low. A “burst growth” in ice crystal formation was observed at *Cn-isoAFP* protein concentrations higher than 0.5 mg/mL (Figure S6). Morphological patterns of single ice crystals grown in solutions containing *Cn-isoAFP* were similar to those obtained in solutions containing Cn-AFP [26]. Therefore, *Cn-isoAFP* appears to have similar biochemical characteristics and antifreeze activity to that of Cn-AFP.

The protein structures of AFPs have been actively investigated to identify ice-binding motifs and determine ice controlling mechanisms [6,10,11,49,50]. However, little is known about the structure of AFPs from diatoms. Through protein modeling, *Cn-isoAFP* was predicted to have three β-helical faces (Figure 4). The B-face of *Cn-isoAFP* was very similar to that of TisAFP6, which has the α-helix covered A-face and a flat B-face consisting of β-sheet [5]. The B-face of TisAFP6 is an IBS for its antifreeze activity. It was hence assumed that the B-face of *Cn-isoAFP* may be a potential IBS. To verify the IBSs of *Cn-isoAFP*, several mutant proteins were generated by site-directed mutagenesis through substitution method to have steric structural hindrances conferred by Tyr residues (V100Y, T196Y and V239Y), hydrophobic interruption by Leu residues (T41L, E145L and T232L), and disruption of hydrophobic core by Ser residue (V40S and I213S). As a result, all site-directed mutated proteins had decreased TH value than *Cn-isoAFP*. In case of Cn-AFP [26], except for the G124Y mutant, most of the Tyr residue mutants (T19Y, T41Y, D175Y, T193Y, and T211Y) had decreased TH activity compared to that of Cn-AFP. Therefore, our results suggest that the B-face of *Cn-isoAFP* is an essential region for ice-binding.

The Poisson-Boltzmann solver (APBS) tool was applied to evaluate and compare the electrostatic potentials of the wild-type protein and its mutants, as was done in previous studies [51,52,53]. Overall distribution of positive and negative charges and negative iso-surfaces of *Cn-isoAFP* and the eight mutated proteins were analyzed (Figure 6). The center of the B-face of *Cn-isoAFP* was shown to have large proportions of neutral and positive charges. To further compare amino acid charges between *Cn-isoAFP* and mutated proteins, negative iso-surfaces on the B-face were examined. Similar to the findings of the electrostatic potential results, negative and neutral charges on the B-face of *Cn-isoAFP* were found to interact with the surfaces of ice crystals. Except for the V100Y mutant protein, all mutant proteins demonstrated different patterns of negative iso-surfaces than *Cn-isoAFP*. In particular, I213S had a large proportion of negative surfaces across its B-face, which might be related to the complete elimination of TH activity. In addition, the secondary structure of I213S as analyzed by circular dichroism spectroscopy showed a different spectrum from that of *Cn-isoAFP* (Figure S7). Ile213 was found to be capable of forming hydrophobic interactions with M221 and V231 located on the same β-loop (β6). Hydrophilic substitution of Ser for Ile could (1) disrupt the hydrophobic core of *Cn-isoAFP*, (2) modify the electrostatic potential of the B-face (acting as the IBS), and (3) generate a more irregular β-helix surface. Therefore, we suggest that hydrophobic interactions are crucial molecular forces required to maintain the flatness of the B-face for antifreeze activity.

## 4. Materials and Methods

### 4.1. Cell Growth and Stress Treatments

*Chaetoceros neogracile* was grown in a low-temperature culture room (4 °C, 25 μmol photon m^−2^ s^−1^ continuous light intensity) in modified f/2 medium [25]. In thermal stress treatment, *C. neogracile* cells were cultured at 10 °C at 25 μmol photon m^−2^ s^−1^ light intensity for 0, 0.5, 1, and 2 h. The sample of 0 h was collected at the moment when internal temperature of medium in a control sample tube increased to 10 °C. For high-light (HL, 600 μmol photon m^−2^ s^−1^) stress treatment, cells were incubated for 0.5, 1, 2, or 4 h at 4 °C. After 4 h of HL stress, cells were transferred to normal light condition (25 μmol photon m^−2^ s^−1^) for 2 or 4 h. The cultures were frozen at −20 °C for 20, 40, or 60 min in order to impose freezing stress.

### 4.2. Cloning of the Cn-isoAFP Gene and Phylogenetic Analysis

The *C. neogracile* AFP isoform gene (*Cn-isoAFP*) was amplified by PCR using degenerate primers (Primers #1 and #2 in Table S1) and cDNA as a template. PCR reaction was carried out using *Pfu* polymerase premix (Elpis, Taejon, Korea). PCR conditions were 95 °C for 4 min, followed by 30 cycles of 95 °C for 30 s, 51 °C for 45 s, 72 °C for 1 min, and then 15 min at 72 °C. The 500 bp PCR product was cloned into the T vector (Promega, Madison, WI, USA) and sequenced (Macrogen, Seoul, Korea). The 5′-unknown sequence of the isoform AFP was obtained using a DNA walking kit (Seegene, Seoul, Korea), and the 3′-unknown region was obtained using a 3′-RACE kit (Roche, Basel, Switzerland) following the manufacturer’s instructions. DNA walking primer and 3′-RACE PCR primer sequence information is provided in Table S1.

The amino acid sequence of the *C. neogracile* isoform AFP showed homology with *C. neogracile* AFP (FJ505233) and other psychrophilic organism AFPs. Sequences for AFPs from the following organisms were obtained from the NCBI database: *Navicula glaciei*, AAZ76251; *Fragilariopsis curta*, ACT99634; *Chlamydomonas* sp., EU190445; *Typhula ishkariensis*, AB109748.1; *Flammulina populicola*, ACL27144; *Lentinula edodes*, ACL27145; *Leucosporidium antarcticum* ACX31168; *Leucosporidium* sp. AY30 ACU30807; *Psychromona ingrahamii*, ZP 01349469.1; *Colwellia* sp., DQ788793; *Deschampsia antarctica*, FJ663038; *Lolium perenne*, FJ663045. Sequence alignment was performed using ClustalW, and the phylogenetic tree was constructed with MEGA5 using the neighbor-joining algorithm [54].

### 4.3. Identification of the Predicted Promoter Sequence

Inverse PCR was carried out to identify the 5′-upstream sequence of *Cn-isoAFP*. Genomic DNA (gDNA) of *C. neogracile* was extracted following the procedure outlined in Gwak et al. [25]. Genomic DNA (0.1 µg) was digested with *Eco*RV for 2 h at 37 °C. The digested gDNA was ligated by T4 DNA ligase (Thermo Fisher Scientific, Waltham, MA, USA) for 1 h at room temperature (25 °C). Ligated DNAs were amplified by PCR with *Cn-isoAFP* inverse PCR primers (Table S1, #7 and #8) and Dream Taq (Thermo Fisher Scientific, Waltham, MA, USA). PCR conditions were as follows: pre-denaturation at 95 °C for 4 min; 30 cycles at 95 °C for 30 s, 63 °C for 45 s, 72 °C for 1 min; and elongation at 72 °C for 10 min. Then, the PCR products were ligated into a T-vector (Promega, Madison, WI, USA). This cloned vector was subsequently sequenced (Macrogen, Seoul, Korea). The 5′-upstream sequence of *Cn-isoAFP* was analyzed by several promoter prediction programs: PLACE [28], PlantCARE [29], and PlantPAN [30].

### 4.4. Sourthern Blot and Northern Blot Assay

Purified gDNA of *C. neogracile* (10 μg) was digested with *Eco*RV, *Kpn*I, and *Xba*I, separated on a 0.8% agarose gel, and transferred to a Hybond™-N^+^ membrane (Amersham, Dayton, TN, USA). Genomic Southern blot was conducted by standard protocols using the radiolabeled *Cn-isoAFP* gene sequence as a probe [55].

Total RNA of *C. neogracile* was extracted using a Plant RNeasy mini kit (Qiagen, Hilden, Germany). RNA (10 μg) was incubated at 65 °C for 5 min for denaturation and loaded on an agarose gel (1.2% agarose, 10% 10X MOPS, and 4.75% formaldehyde). The RNA was then transferred to a Hybond™-N^+^ membrane (Amersham, Dayton, TN, USA) and UV-cross linked for 10 min. The *Cn-isoAFP* probe was amplified by 5′-upstream primers specific for *Cn-isoAFP* (Table S1, #9 and #10). Probes were labeled with ^32^P-dCTP and hybridized in hybridization buffer (5X SSC, 5X Denhardt’s reagent, 0.5% SDS, 100 μg/mL herring sperm DNA) at 62 °C. The membrane was washed twice with wash buffer (2X SSPE and 0.1% SDS) for 25 min. The washed membrane was exposed to an X-ray film and developed.

### 4.5. Cloning for Expression of Recombinant Proteins

To obtain *Cn-isoAFP* pre-mature and mature genes, i.e., genes with or without a signal sequence, respectively, gDNA of *C. neogracile* was amplified by two primer sets. *Cn-isoAFP* ORF with the signal sequence and the *Cn-isoAFP* coding region without the signal sequence were amplified by primers #11 and #13 and #12 and #13, respectively (Table S1). The 5′-forward primer contained the *Kpn*I restriction site, and the 3′-reverse primer included a *Hind*III site (restriction enzyme sites in the primers are underlined in Table S1). *Cn-isoAFPs* were inserted into the pColdI vector (Takara, Kyoto, Japan). Recombinant *Cn-isoAFP* proteins were produced following the instructions in the pCold induction manual (Takara, Kyoto, Japan). Induced cells were collected by centrifugation and re-suspended in 20 mM Tris-HCl buffer (pH 9.0). *E. coli* cells were lysed by sonication (5 s pulse and 10 s delay for 90 s) on ice. Cell pellets were harvested by centrifugation (10,000× *g* for 30 min at 4 °C), and the supernatant was discarded. Pellets were re-suspended in 20 mM Tris-HCl buffer (pH 9.0) and sonicated again (3 s pulse and 5 s delay for 1 min, 4 °C). These sonicated samples were centrifuged, and the supernatants were collected. The supernatants were purified by a His-tag affinity column (Qiagen, Hilden, Germany). The final purified *Cn-isoAFP* recombinant proteins were concentrated using a Centricon filter (Millipore, Bedford, MA, USA).

### 4.6. Antifreeze Activity Assay

Ice crystal morphology was observed using a photomicroscope system consisting of an Olympus BX35 photomicroscope equipped with an ethyl alcohol type temperature controller (Otago nanoliter-osmometer, Dunedin, New Zealand) and a CCD camera. A droplet (approximately 0.5 μL) of the sample solution was frozen and then heated by manipulation of the temperature controller until a single ice crystal was observed separately in the solution. Antifreeze activity was analyzed following the method of Gwak et al. [25]. Purified recombinant Cn-isoAFP protein was concentrated up to 5 mg/mL and then serially diluted to assess its antifreeze activity. A thermal hysteresis measurement was conducted more than three times to obtain precise values.

### 4.7. Structure Prediction of Cn-isoAFP by Homology Modeling

Homology modeling was performed in order to predict the protein structure of Cn-isoAFP. The amino acid sequences of the AFPs of other organisms with high structural homology to Cn-isoAFP were selected and aligned using the PSIPRED program [56]. 3VN3 (antifreeze protein from the snow mold, *Typhula ishikariensis*) was used as a template to model *Cn-isoAFP* in the Phyre2 program [57]. Homology models were generated using Modeller version 9.12 [58], and the best model was selected by analyzing the Modeller Objective Function scores. The best model for Cn-isoAFP was visualized using PyMOL v.1.3 (PyMOL Molecular Graphics System, Version 1.3 Schrödinger, LLC).

### 4.8. Site-Directed Mutagenesis of the Cn-isoAFP

Mutants of *Cn-isoAFP* were produced by site-directed mutagenesis using mutagenic primers (Table S2). To identify residues involved in the IBS, three mutant proteins were generated (V100Y, T196Y, and V239Y; #1 to #6 in Table S2). T41, E145, and T232 of *Cn-isoAFP* were changed to leucine to determine if this decreased the affinity of the protein for ice lattice oxygen atoms (T41L, E145L and T232L; #7 to #12 in Table S2). To confirm the importance of the hydrophobic core of *Cn-isoAFP* for antifreeze activity, V40 and I213 were replaced with serine (V40S and I213S; #13 to #16 in Table S2). All mutated genes were amplified using the gene without the signal peptide sequence (mature form of *Cn*-isoAFP) as a template. PCR was performed using *Pfu* polymerase premix (Elpis, Taejon, Korea). PCR conditions were 95 °C for 4 min; 95 °C for 30 s, 55 °C for 1 min, 72 °C for 5 min, 16 cycles; and 72 °C for 15 min. The amplified DNA was digested with *Dpn*I and ligated into the pColdI vector (Takara, Kyoto, Japan) digested with the same enzymes. The mutated nucleotides were verified by DNA sequencing (Macrogen, Seoul, Korea). Transformation, expression, and purification of each plasmid with a mutation were performed as described above.

### 4.9. Evaluation of Structures of Cn-isoAFP and Its Site-Directed Mutants

The overall structures of *Cn-isoAFP* and its mutants were predicted using circular dichroism (CD) analysis. Far-UV CD was performed at 293 K on a Chirascan CD spectrometer (Applied Photophysics, Leatherhead, UK) between 200 and 260 nM using a 1-cm-pathlength cell. Five scans were recorded; baseline spectra were subtracted from averaged spectra, followed by smoothing of the data.

To analyze the electrostatic fields of *Cn-isoAFP* and its mutated proteins, the adaptive Poisson-Boltzmann Solver (APBS) was adopted in the PyMOL program [42]. The electrostatic field of the proteins was demonstrated by setting the positive and negative iso-surfaces to −1 and 1 kT/e, respectively.

## 5. Conclusions

In summary, we identified the *Chaetoceros neogracile* isoAFP (*Cn-isoAFP*) gene and characterized its biochemical properties. *Cn-isoAFP* was shown to be closely related to the AFPs of other psychrophilic organisms, especially sea ice diatoms. Lowering the freezing temperature below the melting temperature is likely the main function of *Cn-isoAFP* and is facilitated by generation of a protein hydrophobic core and a flat β-helix surface as the ice-binding surface.

## Supplementary Materials

The following are available online at www.mdpi.com/1660-3397/15/10/318/s1, Figure S1: *C. neogracile* AFP isoform nucleotide and amino acid sequence. Under line indicated the signal peptide and star marks exhibit the possible glycosylation site. The red color letters show the N-myristoylation site, Figure S2: Alignment of *C. neogracile* AFP and AFP isoform. The alignment was carried out by ClustalW method. The Black squares show a consensus sequences. The identity of these two sequences is 74.8%, Figure S3: Genomic Southern blot analysis. The gDNA of *C. neogracile* digest with *EcoRV*, *KpnI* and *XbaI*. The *Cn-isoAFP* ORF gene was used as probe. The DNA size markers are shown to left side. E; *EcoRV*, K; *KpnI*, X, *XbaI*, U; Uncut gDNA, Figure S4: Multiple alignments of *Cn-isoAFP* with other AFP, IBP and IRIP of psychrophilic organisms. The multiple alignments were produced by Clustal W, and black squares revealed consensus regions. AFP; antifreeze protein, IBP; ice binding protein, IAFP; ice antifreeze protein, IRIP; ice recrystallization inhibition protein, Figure S5: Phylogenetic tree of selected AFPs, IAFP, IRIP, or IBP amino acid sequences from psychrophilic organisms. The phylogenetic tree produced by MEGA5 and Neighbor-joining method. Bootstrap values obtained with 5000 repetitions. IBP; ice-binding protein, IAFP; ice antifreeze protein, IRIP; ice recrystallization inhibition protein, Figure S6: Ice crystal morphology of *Cn-isoAFP* and its mutant proteins under various protein concentration. The scale bar indicated 100 μm, Figure S7: Circular dichroism spectroscopy of purified Cn-isoAFP and its mutants. Each spectrum is the average of five scans. A correction was made by subtracting the spectra obtained in the presence of buffer only, Table S1: The primer information used in this study. The underline showed a restriction enzyme site, Table S2: Information of site-directed mutagenesis primers. The bold letters indicated the site-directed mutation sequences.
