# Supplementary material for: Identification and Characterization of an Isoform Antifreeze Protein from the Antarctic Marine Diatom, Chaetoceros neogracile and Suggestion of the Core Region
Source: Mar Drugs. 2017 Oct 18;15(10):318. doi: 10.3390/md15100318 (PMC5666426; doi:10.3390/md15100318)
Supplement: Supplementary file 1 [file marinedrugs-15-00318-s001.pdf]

Supplementary data

Supplementary Figure 1. *C.neogracile* AFP isoform nucleotide and amino acid sequence. Under line indicated the signal peptide and star marks exhibit the possible glycosylation site. The red color letters show the N-myristoylation site.

Supplementary Figure 2. Alignment of *C.neogracile* AFP and AFP isoform. The alignment was carried out by ClustalW method. The Black squares show a consensus sequences. The identity of these two sequences is 74.8%.

Supplementary Figure 3. Genomic Southern blot analysis. The gDNA of *C.neogracile* digest with *EcoR*V, *Kpn*I and *Xba*I. The *Cn-isoAFP* ORF gene was used as probe. The DNA size markers are shown to left side. E; *EcoR*V, K; *Kpn*I, X, *Xba*I, U; Uncut gDNA.

Supplementary Figure 4. Multiple alignments of Cn-isoAFP with other AFP, IBP and IRIP of psychrophilic organisms. The multiple alignments were produced by ClustalW, and black squares revealed consensus regions. AFP; antifreeze protein, IBP; ice binding protein, IAFP; ice antifreeze protein, IRIP; ice recrystallization inhibition protein.

Supplementary Figure 5. Phylogenetic tree of selected AFPs, IAFP, IRIP, or IBP amino acid sequences from psychrophilic organisms. The phylogenetic tree produced by MEGA5 and Neighbor-joining method. Bootstrap values obtained with 5,000 repetitions. IBP; ice-binding protein, IAFP; ice antifreeze protein, IRIP; ice recrystallization inhibition protein.

Supplementary Figure 6. Ice crystal morphology of Cn-isoAFP and its mutant proteins under various protein concentration. The scale bar indicated 100 μm.

Supplementary Figure 7. Circular dichroism spectroscopy of purified Cn-isoAFP and its mutants. Each spectrum is the average of five scans. A correction was made by subtracting the spectra obtained in the presence of buffer only.

Supplementary Table 1. The primer information used in this study. The underline showed a restriction enzyme site.

Supplementary Table 2. Information of site-directed mutagenesis primers. The bold letters indicated the site-directed mutation sequences.

Supplementary Figure 1.

																		*	*	*	*	
1	TTT	CAA	AAA	AAG	ACA	GAA	AAA	GAA	GAT	AAA	ATG	AGT	TTC	ATC	AAA	TTT	AAT	CAG	ACC	CTC	60
											M	S	F	I	K	F	N	Q	T	L	
61	GTT	ACG	ACT	GCA	TTG	CTA	GTC	ACT	GCC	GTG	ATA	CTG	CTA	CTA	GGC	GTA	CCA	ATG	ACT	GAA	120
	V	T	T	A	L	L	V	T	A	V	I	L	L	L	G	V	P	M	T	E	
121	GGA	ATC	CTT	CAA	GAG	AAA	CAT	GGA	AAT	CTG	AGG	CGT	CAG	CTC	GAT	GCT	GAA	CCT	TCT	CCG	180
	G	I	L	Q	E	K	H	G	N	L	R	R	Q	L	D	A	E	P	S	P	
181	CCA	CAA	TCT	CGT	GTT	AAG	CTG	CTA	ACT	GCA	GGA	AAG	TTT	GCT	GTT	CTG	TCG	AAA	ACA	GGC	240
	P	Q	S	R	V	K	L	L	T	A	G	K	F	A	V	L	S	K	T	G	
241	GTG	ACG	ACA	ACT	GGT	ACA	ACA	GGC	GTG	ACT	GGT	GCC	ATG	GGA	ACA	AGC	CCC	ATC	TCT	GCT	300
	V	T	T	T	G	T	T	G	V	T	G	A	M	G	T	S	P	I	S	A	
301	ACG	GCG	ATG	ACG	GGA	TTC	GGA	TTG	ATA	ATG	GAC	TCT	GGT	AAC	GCT	TTC	TCG	ACG	TCC	ACT	360
	T	A	M	T	G	F	G	L	I	M	D	S	G	N	A	F	S	T	S	T	
361	CTT	GTG	TCG	GGC	AAT	GTT	TAT	GCA	GCC	GAC	TAT	GAA	TCT	CCC	ACG	CCC	AAC	ATG	CTG	ACA	420
	L	V	S	G	N	V	Y	A	A	D	Y	E	S	P	T	P	N	M	L	T	
421	GTA	GCA	GTC	CTC	GAC	ATG	CAG	GGC	GCA	TAC	GTC	GAT	GCT	GCA	GGT	CGC	CCC	GAC	CCA	GAC	480
	V	A	V	L	D	M	Q	G	A	Y	V	D	A	A	G	R	P	D	P	D	
481	TAT	GCA	GAC	CTC	GGC	GCT	GGA	AGC	ATT	GAG	GGT	TTA	ACT	CTC	GAT	CCT	GGC	CTG	TAC	AAG	540
	Y	A	D	L	G	A	G	S	I	E	G	L	T	L	D	P	G	L	Y	K	
541	TGG	GGG	ACA	AAT	GTC	GAA	CTC	ACC	AGC	AGC	CTC	ACC	TTC	AAT	GGT	TCT	AGC	ACG	GAC	ATC	600
	W	G	T	N	V	E	L	T	S	S	L	T	F	N	G	S	S	T	D	I	
601	TGG	ATC	TTA	CAG	ATC	GGC	GGA	GAT	GTA	AAG	GTA	GGC	AGC	GGT	GCA	ATC	GTT	GAA	CTC	ACT	660
	W	I	L	Q	I	G	G	D	V	K	V	G	S	G	A	I	V	E	L	T	
661	GGT	GGT	GCC	TTG	GCA	GAA	AAC	ATT	TTC	TGG	CAG	ATC	GCA	GGC	AAG	ACT	ACT	CTC	GGC	ACC	720
	G	G	A	L	A	E	N	I	F	W	Q	I	A	G	K	T	T	L	G	T	
721	TCA	TCC	CAT	GTA	GAG	GGT	GTT	TTC	CTT	TGC	AAT	ACA	CAA	ATC	GCA	TTC	GAA	ACC	GGA	AGC	780
	S	S	H	V	E	G	V	F	L	C	N	T	Q	I	A	F	E	T	G	S	
781	AGT	ATG	AAT	GGA	GCT	GCA	CTG	GCA	CAG	ACG	GCA	GTG	ACA	CTG	GAT	GCT	GCT	ACC	ATT	GTC	840
	S	M	N	G	A	A	L	A	Q	T	A	V	T	L	D	A	A	T	I	V	
841	AAG	ACT	TCG	GTG	TGT	GAC	GCC	ACT	GTC	GGG	TGT	GTG	AAA	GAC	TAA	TTT	GCT	GAT	GAA	TCC	900
	K	T	S	V	C	D	A	T	V	G	C	V	K	D	*						
901	TTA	CCA	AAG	CAG	ACA	GAG	GAT	AAG	CGC	AAA	TAC	ACA	TCT	ACT	ATT	ACA	GTA	ATA	CAT	CAT	960

Supplementary Figure 2.

```
Consensus  MS. I . . N. TLV. TALL. . AV. . LLGVPMAEG. . QEK. G. LRRQLD. EP. . . . S. VKLLTAG. FA. L. KTGVTTTG. T. . . G. MGTSPI . . . A. TGFGLI .
Cn-isoAFP  MSFIKFNQTLVTTALLVTAVILLLGVPMAEGILQEKHGNLRRQLDAEPSPPQSRVKLLTAGKFAVLSKTGVTTTGTTGVTGAMGTSPISATAMTGFGLIM
Cn-AFP     MSLITINHTLVVTALLFAAVA-LLGVPMAEGLRQEKRGGLRRQLDDEP---LSAVKLLTAGRFAILTKTGVTTTGPTDLKGDMGTSPITGAAITGFGLIT

Consensus  D. S. . . FSTS. LV. G. V. A. DY. SPTPNMLTVAVLDMQ. AYVDAAGRPDPDY. . LGAG. I EGLTL. PGLYKWGT. V. FT. SLTF. GS. TDI W LQI DGDV
Cn-isoAFP  D-SGNAFSTSTLVSGNVYAADYESPTPNMLTVAVLDMQGAYVDAAGRPDPDYADLGAGSIEGLTLDPGLYKWGTNVEFTSSLTFNGSSTDIWLQIDGDV
Cn-AFP     DPSDTTFSTSSLVTGQVFASDYTSPTPNMLTVAVLDMQAAYVDAAGRPDPDYVELGAGNIEGLTLEPGLYKWGTDVGFTNSLTFDGSDTDIWLQIDGDV

Consensus  . . GSGA. V. L. . . A. A. NI FWQI AGKT. LGT. SHVEGVFLC. T. I . F. TGSSMNGAALAQTAVTLD. ATI VK. SVCD. . VGCV. . . -
Cn-isoAFP  KVGSGAIVELTGGALAXNIFWQIAGKTTLGTSSHVEGVFLCNTQIAFETGSSMNGAALAQTAVTLDAATIVKTSVCDATVGCVKD.
Cn-AFP     TAGSGAKVKLINDAKAENIFWQIAGKTDLGTTSHVEGVFLCSTAITFKTGSSMNGAALAQTAVTLDSATIVKESVCDVDVGCVAPN.
```

Supplementary Figure 3.

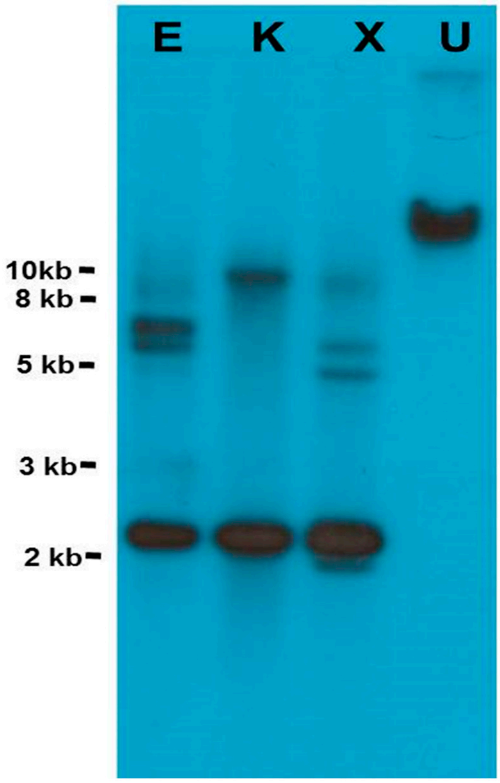

Supplementary Figure 4.

```
C. neogracile isoAFP     MSFIKFNQTLVTTALLVTAVILLLGVPMTEGILTEKHGNLRRQLDAEPSPPQSRVKLLTAGKFAVLSKTG------VTTT  74
C. neogracile AFP        MSLITINHTLVVTALLFAAVA-LLGVPMAEGHRQEKRGGLRRQLDDEP---LSAVKLLTAGRFAILTKTG------VTTT  70
N. glaciei IBP           ----MMAKTVTVAVASSVAAS-------------AVDG--------------------TAG--DAVSKAG------VSTT  35
T. ishikariensis AFP     -----MSASSAVAASSVSAAG-------------SAVG--------------------TAG--NYVASTG------VSTV  34
P. ingrahamii IBP        MNTKNYSKKSSSGVGNAVAG--------------YSVN--------------------SAD--NAVSKSG--------TN  36
F. curta IAFP            --------GMSVASASSTAVN-------------RTAG--------------------------DVAKAG-------TNV  26
F. populicola IBP        --------MTSSVGSSSVAG---------------AVG--------------------KAN--ASTGVSN--------VD  27
Colwellia sp. IBP        MKTSN-SKKVMGSAGNVMAAG-------------YAVG--------------------AG----TTSKSG--------TD  34
L. edodes IBP            MSTTN----TSGAVVSVVAAG-------------GSVG--------------------SGG--GSGGGSGGGNGGGGAN  41
Luecosporidium sp. AFP   --------MSSTGAGGGVNGR-------------DSVG--------------------VASN-AAKAGSS--------VD  30
L. antarcticum AFP       --------MRSNHAASVRCAH------------------------------------------SRRTDSS-------SST  23
Chlamydomonas sp. IBP    MSSSMKAAACMATSMAAVVCK------------MDADG--------------------NTTAACSVGVSG------GGSG  42
D. antarctica IRIP       -----MAKCGSAAASATACHSR------------DRAGARNGG---------------VGGVRAAWSGDG-------CCD  41
L. perenne IRIP          ------MKSWAAASVAVSCHD-------------DARGAGNS----------------NGGVHAKW--DN-------CCS  36

C. neogracile isoAFP     GTTGVTGAMGTSPISATAMTGFGLIMD-SGNAFSTSTLVSGNVYAADYESPTPNML---TVAVLDMQGAYVDAAGRPDPD  150
C. neogracile AFP        GPTDLKGDMGTSPITGAAITGFGLITDPSDTTFSTSSLVTGQVFASDYTSPTPNML---TVAVLDMQAAYVDAAGRPDPD  147
N. glaciei IBP           GTVTGDGTSASTATGAKDSS--N----------TSTSSVTGK-YAADYTATSK------MTTASDMSTAT-DAAGRSDD-  94
T. ishikariensis AFP     SVTGAVGVSGTAASTGSSGT--G----------TSTSSVTG--TGADYGTTS--------TTAGDMGTAY-NAATRSGD-  90
P. ingrahamii IBP        VSDSVTGDVG------------------ASTGAATTCSVVG-DYATTANGACSNAG---TGSAGDMGAYN-DAAGRVSD-  92
F. curta IAFP            GGVTGDGVSAASAMTGDMD------------SSNSTSVVDGSVYANYMSTGTKT------TAVSDMTAYN-DAAARVTS-  86
F. populicola IBP        SSVNCDGVSGASGVTGSTG----------DSGGSSTSKVTGRVYASTYGDTAS-------TTAVDMNAYK-DARDDTNH-  88
Colwellia sp. IBP        VYSTVTGNVG------------------TSTGAAN-CDVTG-AMYTVDSAGCSNS-----YAVSDMGAYN-DAAGRVADH  88
L. edodes IBP            AGAAVNGTAGNYAAKSGSTVSSG--NGVSSTTATGSTDSTGKATSVVGAASAATTT---TTAVSDMTA-N-DATGRVTDT  114
Luecosporidium sp. AFP   SAG--DGVSAAATYTGGT-----------DSSTTYATSVTG--YAADYSTTNY-------AAAVANATAY-NAAGVDDG-  86
L. antarcticum AFP       SAATADGVAGYDVVAR-----------------SATGAATG--NVGSGSTATG--------TVVDHG-------------  63
Chlamydomonas sp. IBP    GSVSYTDANNRSTCGDDD----------VDSSYSSTNNCRGCSNASNTTSTRSTACTSKYGCMAVVGAATTDWSSSANTT  112
D. antarctica IRIP       WGVGCDGASGRVTTT----------------RGAGG-ASAGVHVKGNRRTANRSG------TNNSVR-------GR----  87
L. perenne IRIP          WGVGCDGGSGRVTTWG---------------HGAGHTASAGARSNANNKVGTSWGVDHCYDSNNSVGKNRRCNVGRSGMA  101

C. neogracile isoAFP     Y-----ADLGAGSIEGLTLDPGLYKWGT-------------NVELTSSLTFNGSSIDIWI-L-QIGGDVKVGSGAIVELT  210
C. neogracile AFP        Y-----VELGAGNIEGLTLEPGLYKWGT-------------DVGFTNSLTFDGSDIDIWI-L-QIDGDVTAGSGAKVKLI  207
N. glaciei IBP           ------G-AGSGTVAG-------YKWGT-------------DVSTSS---VDGSATDVWV-A-KDVGNGAMYTGTAKAN-  141
T. ishikariensis AFP     ------Y-TGAGGTTG-------YKWTS-------------SVGASAD--TSGTSTDTWD-G-TGAAGKKTVGGAAKNVW  139
P. ingrahamii IBP        ---------TGAGGGT------GYKWS---------------TDVGTSDVTKGNATDVWA-G-TSAAYKNVTAGGAANVW  141
F. curta IAFP            ---------GGCAGAG-------ATYTN-------------GAGGGTTGVYTYTDVRTDSDVTDGKNDSSVKTSKNAANT  137
F. populicola IBP        --------TGAGGAVG-------YKTTG--------------VSTADVT--GGTDTYAG-----TSAAGVKNVGGANVVW  132
Colwellia sp. IBP        --------TGTGGGTG------VYKWS---------------SDVNSTDVTNGTMDDVWMSG-NNANAKRVTTGGAAKNW  138
L. edodes IBP            --------NGGGGGVT------GYKWTG-------------AVSVNSTGVTAGTDH-----A-TGAAASRVTVGGASNVW  161
Luecosporidium sp. AFP   --------AGR-DTVG-------YKWTS-------------SVSVTDTG--NGDATWVAG----GSADGVATAGGANSTN  131
L. antarcticum AFP       --------TCSAG----------VKYCG-------------ADSSTSATSVKGRDADS--------------SAGAATDV  98
Chlamydomonas sp. IBP    STCASSATSGRVGGAARNDSGAMTAVVKGSTTSTACDGGAHSANASTTDSTTGTAVDGGAVVGTNATVASVKSSTTSSTA  192
D. antarctica IRIP       -------NNAAGN-------------DN-------------TVSGNNNTVS-GSNTVVGSDN---TGSKHVVSGRKHVTD  130
L. perenne IRIP          STNMTVKHNASGNTTG-TNNYVRSGVNN-------------VVSGNHNTVTSGNNNVVSGNHNTVSGTNHVVTGNNHVVT  167

C. neogracile isoAFP     GGALAENIFWQIAG-KTTLGTSSHVEGVFLCNTQIAFETGSSMNGAALAQTAVTLDAATIVKTSVCDATVGCVKD.      285
C. neogracile AFP        NDAKAENIFWQIAG-KTDLGTTSHVEGVFLCSTAITFKTGSSMNGAALAQTAVTLDSATIVKESVCDVDVGCVAPN.     283
N. glaciei IBP           -----------VSG-AVNGTTAHVGN-------SATAATGSSNGKASTATDSVTVS.                        179
T. ishikariensis AFP     -----V-----VAG-AVNVGAKGTAK-------TAVTKTGSSNGRA-TAVASATVK.                        177
P. ingrahamii IBP        -----------VAD--SVTGTGAHGG--------KAVNTGATVKGRATAVTKNKTAVSHSVNNS.                185
F. curta IAFP            ---------DVTNGAKANWSVAVNGAGAHMKGVATAVKTGSNVGRVAATAVTATTDTAATSSTR.                193
F. populicola IBP        -----------AVADSVTVAATSSGG------KTVVVNTNASVGRATAVVKATVVGVCGA.                    176
Colwellia sp. IBP        -----------VAG--YTAGTYASGVS------KTSVNTGTTVNGRATAVTKNTNATYA.                     179
L. edodes IBP            -----------AATSVVTAGAGSHGVVA-----KTAVTTGATMNGRATVASATVG.                         201
Luecosporidium sp. AFP   -----------AVGDDVTVGKGAHG-------VAKRVTTGSSNGRVSTVAKATVNSVAVVKRSNARW.             181
L. antarcticum AFP       ----------------VAAWKSASS----------ADYTKRDAGGSDTAG.                              123
Chlamydomonas sp. IBP    VTTG-----GMVAGGAVSDDSTTNGDAKGKCVWDKCSYTDKTNNTWTGCAKSSWCKAHDGNKWTTCGMGRCY.         260
D. antarctica IRIP       -----------NNNKVSGNDNNVSGSH-------TVSGSHNTVSGSNNTVSGSNHVVSGSNKVVTGG.             180
L. perenne IRIP          -----------RNNTVSGSHHKVSGGHN------TVSGSHNTVSGSHNTVSGSNHVH-GNNKVVTGG.             217
```

Supplementary Figure 5.

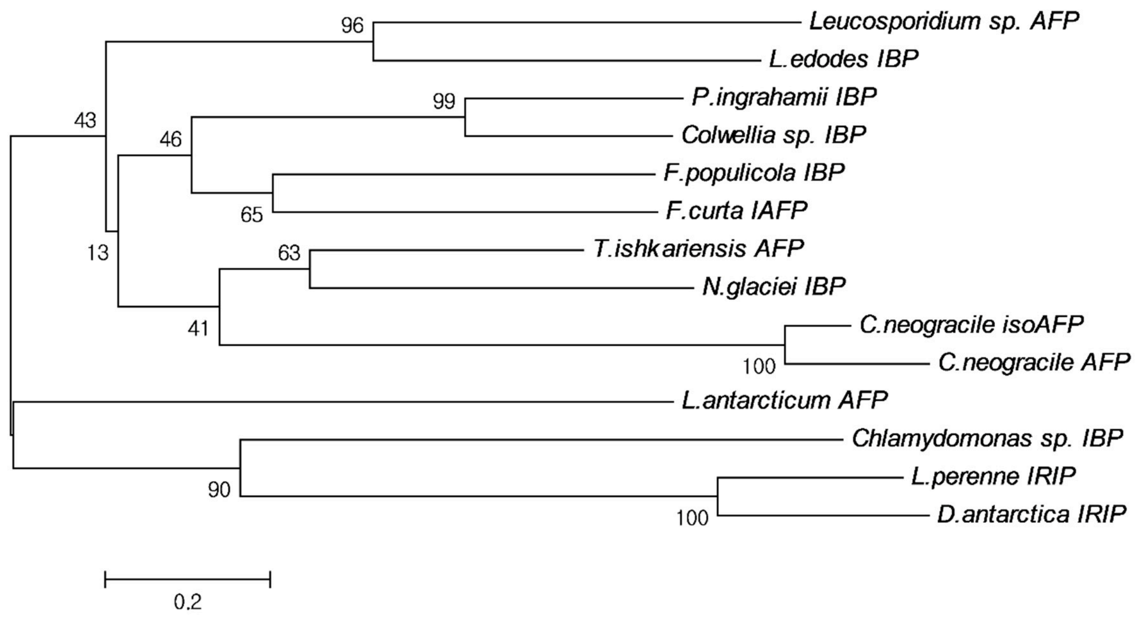

Supplementary Figure 6.

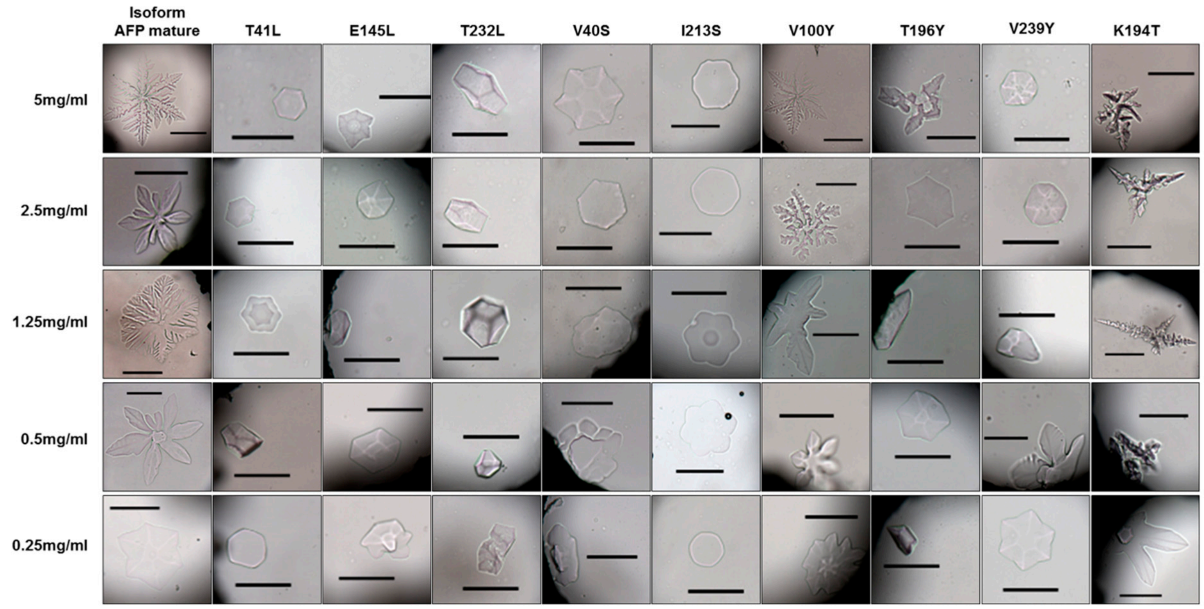

Supplementary Figure 7.

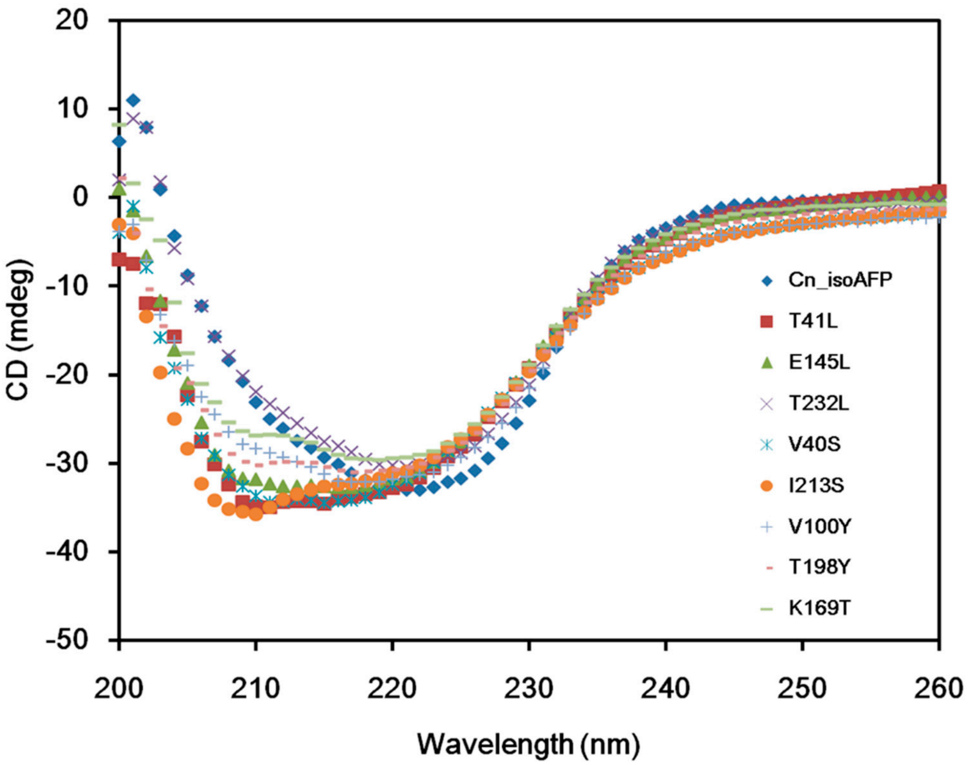

Supplementary Table 1.

Number	Primer name	Sequences (5' → 3')	Tm (°C)
1	AFP degenerated forward primer	AAR CAN GGN GTN CAN CAN AC	54
2	AFP degenerated reverse primer	ARN GTN CAN GCN GTY TGN GC	56
3	Isoform AFP DNA walking target specific primer 1	AGA GTA GTC TTG CCT GCG AT	57
4	Isoform AFP DNA walking target specific primer 2	GGA TCG AGA GTT AAA CCC TC	57
5	Isoform AFP DNA walking target specific primer 3	CCA TTA TCA ATC CGA ATC CCG T	58
6	Isoform AFP 3' race primer	AAC ATT TTC TGG CAG ATC GC	56
7	Isoform AFP inverse primer 1	CAC GGC AGT GAC TAG CAA TGC	63
8	Isoform AFP inverse primer 2	ATG GAG CTG CAC TGG CAC AGA	63
9	Isoform AFP 5' UTR probe forward primer	TGA GTT TAG GTC CAG CGT CCG	63
10	Isoform AFP 5' UTR probe reverse primer	GGT ACG CCT AGT AGC AGT ATC	63
11	Isoform AFP pCold pre-mature forward primer	GGT ACC ATC CTT CAG GAG AAA	59
12	Isoform AFP pCold mature forward primer	GGT ACC ATG AGT TTC ATC AAA TTT	58
13	Isoform AFP pCold reverse primer	AAG CTT TTA GTC TTT CAC ACA CCC	61

Supplementary Table 2.

Number	Primer name	Sequences (5' → 3')	Tm (°C)
1	V100Y forward	CCA CGC CCA ACA TGC TGA CA**T AC**G CAG TCC TCG ACA TGC AGG G	84
2	V100Y reverse	CCC TGC ATG TCG AGG ACT GC**G TA**T GTC AGC ATG TTG GGC GTG G	84
3	T196Y forward	GGC AGA TCG CAG GCA AGA CT**T AT**C TCG GCA CCT CAT CCC ATG	93
4	T196Y reverse	CAT GGG ATG AGG TGC CGA G**AT A**AG TCT TGC CTG CGA TCT GCC	83
5	V239Y forward	CAC TGG ATG CTG CTA CCA TT**T AC**A AGA CTT CGG TGT GTG ACG CC	82
6	V239Y reverse	GGC GTC ACA CAC CGA AGT CTT **GTA** AAT GGT AGC AGC ATC CAG TG	82
7	T41L forward	TTC TGT CGA AAA CAG GCG TG**C TG**A CAA CTG GTA CAA CAG GCG T	81
8	T41L reverse	ACG CCT GTT GTA CCA GTT GT**C AG**C ACG CCT GTT TTC GAC AGA A	81
9	E145L forward	ACA AGT GGG GGA CAA ATG TC**C TA**T TCA CCA GCA GCC TCA CCT T	81
10	E145L reverse	AAG GTG AGG CTG CTG GTG AA**T AG**G ACA TTT GTC CCC CAC TTG T	81
11	T232L forward	CAC TGG CAC AGA CGG CAG TG**C TA**C TGG ATG CTG CTA CCA TTG T	83
12	T232L reverse	ACA ATG GTA GCA GCA TCC AG**T AG**C ACT GCC GTC TGT GCC AGT G	83
13	V40S forward	CTG TTC TGT CGA AAA CAG GC**T CG**A CGA CAA CTG GTA CAA CAG GCG	82
14	V40S reverse	CGC CTG TTG TAC CAG TTG TC**G TC**G AGC CTG TTT TCG ACA GAA CAG	82
15	I213S forward	GGG TGT TTT CCT TTG CAA TAC ACA A**TC C**GC ATT CGA AAC CGG AAG CAG	81
16	I213S reverse	CTG CTT CCG GTT TCG AAT GC**G GA**T TGT GTA TTG CAA AGG AAA ACA CCC	81
